# Inferring pulmonary exposure based on clinical PK data: accuracy and precision of model-based deconvolution methods

**DOI:** 10.1007/s10928-021-09780-x

**Published:** 2021-09-28

**Authors:** Anneke Himstedt, Jens Markus Borghardt, Sebastian Georg Wicha

**Affiliations:** 1 Department of Clinical Pharmacy, Institute of Pharmacy, University of Hamburg, Bundesstrasse 45, 20146 Hamburg, Germany; 2 Research DMPK, Drug Discovery Sciences, Boehringer Ingelheim Pharma GmbH & Co. KG, Biberach, Germany

**Keywords:** Pharmacokinetics, Pulmonary, Target-site exposure, Modeling and simulation, Inhalation

## Abstract

Determining and understanding the target-site exposure in clinical studies remains challenging. This is especially true for oral drug inhalation for local treatment, where the target-site is identical to the site of drug absorption, i.e., the lungs. Modeling and simulation based on clinical pharmacokinetic (PK) data may be a valid approach to infer the pulmonary fate of orally inhaled drugs, even without local measurements. In this work, a simulation-estimation study was systematically applied to investigate five published model structures for pulmonary drug absorption. First, these models were compared for structural identifiability and how choosing an inadequate model impacts the inference on pulmonary exposure. Second, in the context of the population approach both sequential and simultaneous parameter estimation methods after intravenous administration and oral inhalation were evaluated with typically applied models. With an adequate model structure and a well-characterized systemic PK after intravenous dosing, the error in inferring pulmonary exposure and retention times was less than twofold in the majority of evaluations. Whether a sequential or simultaneous parameter estimation was applied did not affect the inferred pulmonary PK to a relevant degree. One scenario in the population PK analysis demonstrated biased pulmonary exposure metrics caused by inadequate estimation of systemic PK parameters. Overall, it was demonstrated that empirical modeling of intravenous and inhalation PK datasets provided robust estimates regarding accuracy and bias for the pulmonary exposure and pulmonary retention, even in presence of the high variability after drug inhalation.

**Supplementary Information:**

The online version contains supplementary material available at 10.1007/s10928-021-09780-x.

## Introduction

One key assumption of pharmacokinetic/pharmacodynamic (PK/PD) analyses is that the local drug concentration at the target site, i.e. the target organ, is driving the efficacy. While determining the local tissue PK might be possible in preclinical experiments [1], adequate determination of the local concentration–time profile in clinical studies is challenging. While there are methods to determine tissue concentrations in humans (e.g. microdialysis [2] or imaging techniques [3]), data based on these methods is rarely available due to the related complexity [2, 4]. Furthermore, more invasive methods may be difficult to justify in routine clinical studies. Therefore, plasma concentration–time profiles are often considered as a surrogate in PK/PD analyses assuming to provide an adequate representation also for the tissue concentrations [5].

For inhaled drugs, high local tissue concentrations and consequently high pulmonary efficacy can be achieved even before drug absorption into the systemic circulation. This also means that directly considering the plasma concentration as a surrogate for pulmonary tissue concentration and pulmonary efficacy might be of limited value. Instead, it is essential to make best use of the plasma PK data to indirectly infer the local pulmonary PK, which can be considered a better surrogate for pulmonary efficacy. In theory, deconvoluting the plasma PK profiles by numerical deconvolution methods (e.g., point-area deconvolution) allows to infer on pulmonary PK [6, 7]. However, these traditional deconvolution methods often assume linear systemic disposition kinetics and / or a single linear (pulmonary) absorption process, which might often not hold true [8]. Instead, model-based deconvolutions can account for these complexities and (pulmonary) absorption models of varying complexity were applied to infer on pulmonary exposure and residence time after oral drug inhalation, which are relevant for the extent and duration of efficacy, respectively [9–11]. These two PK characteristics can subsequently facilitate the comparison between different drugs or inform whether an inhaled drug qualifies for twice daily or even once daily dosing. To perform a (model-based) deconvolution, it is essential to have both data after drug inhalation and after intravenous (i.v.) dosing [12]. However, even having both datasets available, different model structures as well as different approaches combining i.v. and inhalation data in a model building process were published [9, 10, 12–16]. So far, however, a systematic comparison of all available models and whether sequential or simultaneous parameter estimation is best for inhalation PK models is missing. Potentially even more important, it was also never quantitatively evaluated if un-biased and precise inference of the extent of pulmonary exposure and retention time can be achieved based on realistic clinical datasets.

This modeling and simulation study aims at evaluating the overall suitability of PK modeling for inferring the extent and duration of pulmonary exposure based on plasma PK data and, if suitable, identify the best modeling strategy for this purpose. The focus lays on (1) to evaluate the impact of the choice of a pulmonary absorption model on inferring pulmonary exposure, and (2) to compare whether sequential or simultaneous parameter estimation based on i.v. and inhalation PK is meaningful, and (3) to quantify bias and imprecision of the different methods when inferring on extent and duration of pulmonary exposure. To this end, different model structures and modelling strategies were compared based on previously applied clinical studies for inhaled drug programs. Ultimately, this analysis gives insights into what modelling based on clinical data can provide and what the limitations might be.

## Methods

### Investigated pulmonary absorption models

**Fig. 1:**
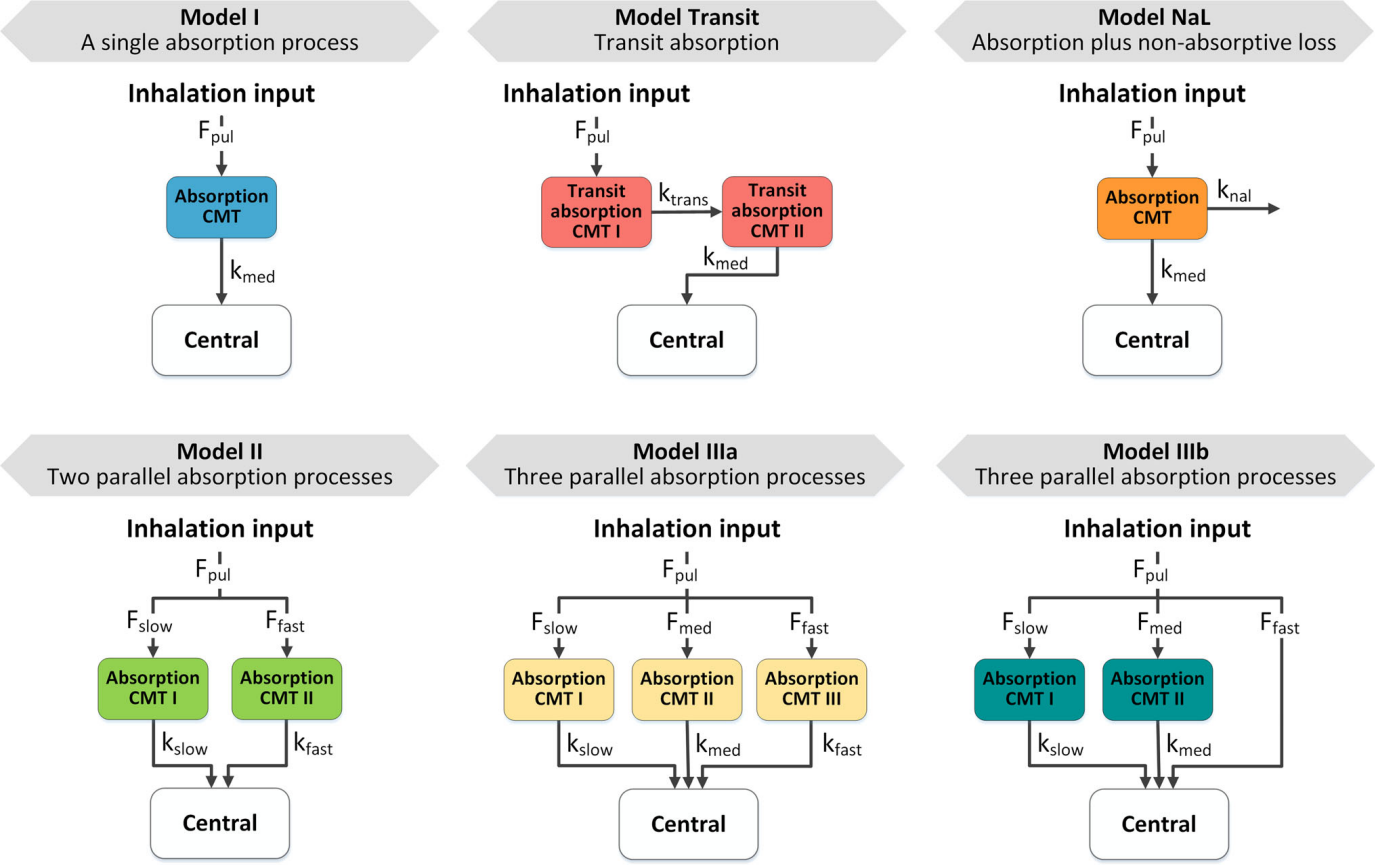
Structural models for pulmonary absorption. Structure and parameterization were based on published models [6, 7, 9–12]. *CMT* compartment, *F*_*Pul*_ pulmonary bioavailability or designated lung dose, *F*_*slow/med/fast*_ fraction of the lung dose slowly/intermediately/fast absorbed, *k*_*slow/med/fast*_ slow/intermediate/fast absorption rate constants, *k*_*trans*_ transit rate constant, *k*_*nal*_ non-absorptive loss rate constant

Models with structurally different pulmonary absorption components were built and parameterized based on the respective publications [9, 12, 14–16], and are shown in Fig. 1. All parameter values used in this study can be found in the supporting information (Supplementary Material S2.2, Table S2). Concomitant absorption of swallowed drug via the gastro-intestinal tract was not accounted for to reduce unnecessary complexity, as this absorption process can be prevented by ingesting active charcoal parallel to drug inhalation in clinical studies [17, 18].

### Evaluation of the structural identifiability of pulmonary absorption models

**Fig. 2:**
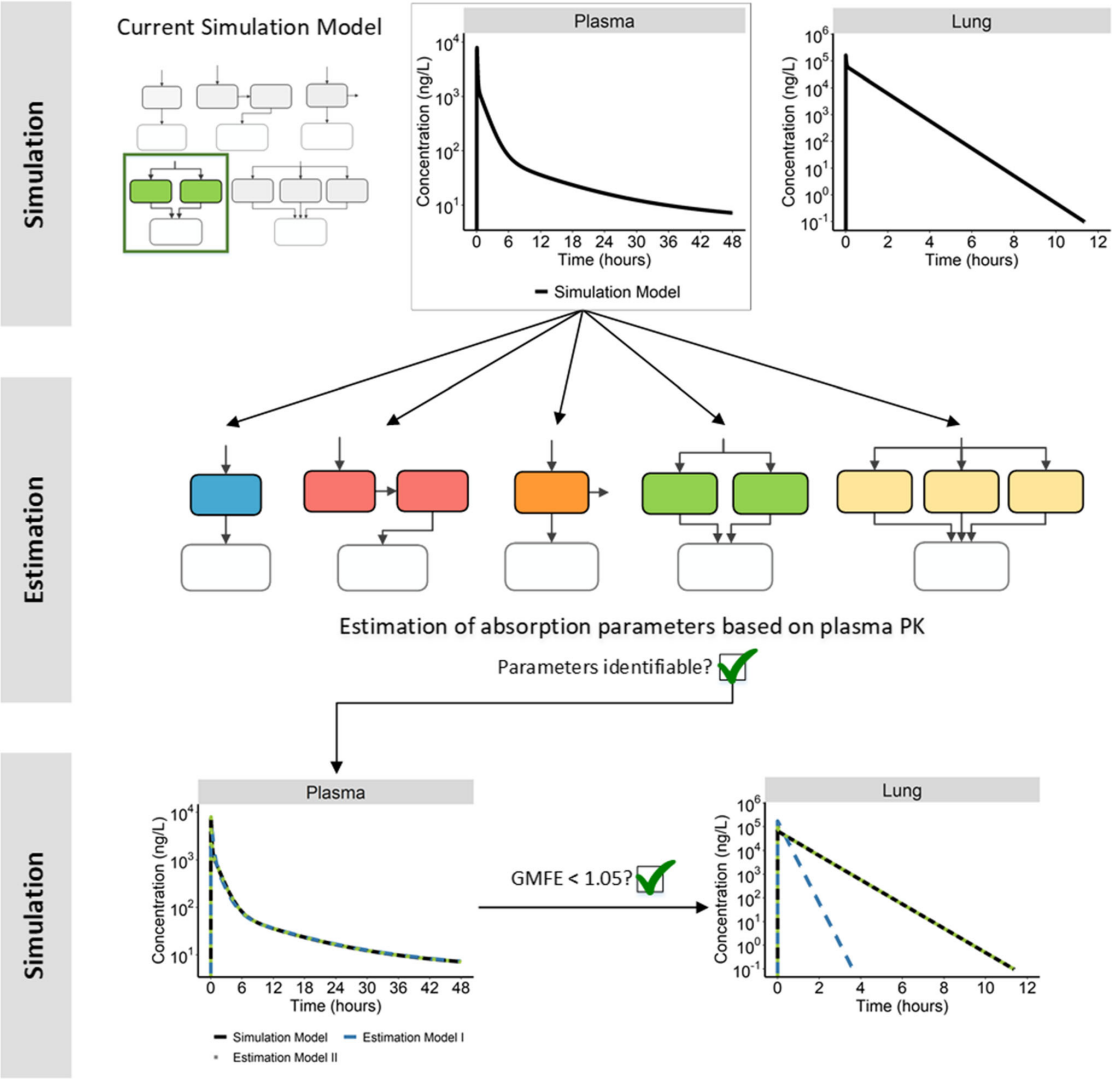
Schematic of structural identifiability evaluation workflow. *PK* pharmacokinetics, *GMFE* geometric mean fold error of the plasma PK profile simulated with the estimation models

A simulation-estimation analysis with models I (a single absorption process), II (two parallel absorption processes), IIIa (three parallel absorption processes), Transit, and NaL (single absorption process with parallel non-absorptive loss) was performed in R (Version 3.2.2) utilizing the package “deSolve” (Version 1.28) [19, 20]. All of these structural models were used to simulate plasma and lung concentration–time profiles over 48 h, resulting in five datasets (one for each model in Fig. 1, except for Model IIIb). To avoid distortion in the identifiability analysis, these profiles were simulated without residual error, which however was included in the second analysis to evaluate the performance of pulmonary absorption models in a clinical trial setting (see below). A lung volume of 0.84 L [21] was assumed to convert unabsorbed amounts to pulmonary concentrations. The models applied in this step will be referred to as the “Simulation Model”. A very rich sampling scheme with concentration data simulated every 0.01 h was selected to rule out the impact of sparse sampling designs and thereby to focus on the structural identifiability between the different models. Afterwards, each of the five models was applied for parameter estimation (“estimation model”) based on the simulated plasma concentration data resulting from each of the Simulation Models. Thus, in total 25 estimation analyses were performed. Since the focus of this part of the work laid on the comparison of pulmonary absorption models, the systemic disposition parameters of the Estimation Models were fixed to the published values and only the pulmonary PK parameters were estimated. If identifiability of the absorption parameters was given based on a non-singular Fisher information matrix and non-infinite standard errors, full plasma and inferred lung concentration–time profiles were generated with the newly estimated parameter values. Both these predictions were compared to the before simulated plasma and lung concentration–time profiles. For each Estimation Model, ten retries were performed to avoid convergence to local minima (Supplementary Material S2.2). Only when plasma equivalence was given (see “evaluation criteria” below), the model-based simulations were further compared with regard to the pulmonary exposure. A schematic representation of this workflow can be found in Fig. 2.

### Link between empiric and mechanistic PK modeling

All empirical models described here consider the pulmonary drug absorption one-directionally, i.e. no back flow from the systemic disposition to the lung is accounted for. To evaluate the potential bias caused by this simplification, all models were additionally fitted to data generated using a semi-mechanistic PK model for salmeterol [1] (Supplementary Material S2.3). This semi-mechanistic model was previously developed with both plasma and lung concentration data and accounted for back-flow from the systemic disposition to the lung. Thus, five additional simulation-estimation analysis were performed, finally resulting in 30 different combinations of the Simulation and Estimation Models.

### Performance of pulmonary absorption models in a clinical trial setting

Models I-III cannot be differentiated based on prior mechanistic understanding of the pulmonary PK. Therefore, these models are often discriminated solely based on their description of the available (plasma and urine) PK data [9, 15]. To explore the performance of these models to infer extent and duration of pulmonary exposure based on real-life clinical datasets, population PK analyses were carried out in NONMEM® Version 7.4.3 (ICON development solutions, Ellicot City, USA). Here, the chosen Simulation Models (‘Models II’ and ‘IIIa’) were reproduced in NONMEM® with the model structure, parameter values for population as well as all variability estimates, number of subjects, and sampling schemes taken directly from the respective publications (Supplementary Material S2.2, Table S2) [9, 15, 22]. As these models were built on some of the richest datasets for PK after both i.v. administration and inhalation published to date, these examples were taken as best-case examples to investigate how meaningful and accurate model-based deconvolution methods can be. Slight adjustments were made to the stochastic part of the original models, i.e., only up to four inter-individual and/or inter-occasional variabilities were included. This was done to prevent selecting a model structure over another model structure only due to a different number of included variability parameters. The residual variability was assumed to be proportional, oral absorption processes for ‘Model II’, and the inter-individual variability on the first proportionality factor (PF1) identified for ‘Model IIIa’ were not included. A summary of the dataset characteristics as provided in the respective publications, including the modifications to the stochastic models made in this study, can be found in Table 1.

**Table 1:** Data summary for the two simulation models used in the population PK approach

	Population analysis I	Population analysis II
Simulation model	II (AZD5423)	IIIa (Olodaterol)
Number of subjects Inhalation (intravenous)	13 (13)	88 (48)
Inhalation and intravenous PK in the same subjects?	Yes	No
Urine data	No	Yes
Type of trial	Single dose (cross-over intravenous and inhalation)	Single dose (intravenous) Single and multiple dose (inhalation)
Systemic PK model	Four compartment model	Four compartment model
Inter-individual variability	F_pul_, CL, V1, Q2	CL, V1, Q2
Inter-occasion variability	–	F_pul_
Estimation models	II, I Three or four compartment systemic model	IIIa, II, IIIb

*CL* systemic clearance, *V1* central volume of distribution, *Q2* intercompartmental clearance to the second systemic compartment

The Simulation Models were used to generate PK datasets after i.v. administration and oral inhalation, this time including residual, inter-individual, and inter-occasion variability. Analogous to the first analysis, related models (parallel absorption models that were proven to be structurally identifiable, see Table 1) were fitted to the simulated plasma concentration–time datasets. The Estimation Models were chosen to evaluate the influence of capturing the right number of absorption processes on the extent and duration of exposure (AUC_0-inf_ for both plasma and lung and t_C24h,lung_). These PK metrics were calculated based on the population parameters. Furthermore, the influence of the systemic model on the same metrics was investigated in the analysis with ‘Model II’ as the Simulation model.

As the i.v. and inhalation study arms for ‘Model II’ (AZD5423) were conducted in the same individuals, the generated PK data from this model was used to compare different modeling approaches: These were.

(i) sequential modeling of i.v. and inhalation data, with either fixed systemic population PK parameters as well as their variance (PPP, theta and omega values estimated in a first step based on i.v. data),
(ii) fixed individual systemic PK parameters (IPP, fixed empiric Bayesian estimates), and
(iii) simultaneous estimation of both systemic and pulmonary PK parameters based on the combined dataset of i.v. and inhalation data (ALL) [23].

Estimation of parameters based on PK datasets generated with ‘Model IIIa’ was done sequentially, using the PPP approach. Here, the individual PK parameters (i.e., the Empirical Bayes Estimates) of the four compartmental systemic model in the inhalation trial could vary within the pre-estimated inter-individual variability. To evaluate the probability of choosing the “right” model, model fits to the same dataset were compared with regard to the Akaike Information Criterion (AIC, [24]).

For all population analyses, the parameter estimation was performed using first-order conditional estimation (FOCE) with interaction. If the estimation step failed, up to two retries with varying initials were performed. The simulation-estimation process was repeated 500 times for each analysis.

### Non-compartmental analysis of simulated datasets

In addition, or instead of analyzing clinical PK data with population approaches, non-compartmental analyses (NCA) [25] are often applied and can be used to infer absorption kinetics. Therefore, model-based predictions were compared to results from the NCA. To infer the pulmonary AUC_0-inf_, the equation for AUC calculation in plasma (Eq. 1) was adjusted to the lung, inserting F_Pul_ as the bioavailability (F) and the pulmonary absorption rate k_a_ as the elimination rate from the lung:

1 $$AU{C}_{0-Inf}= \frac{Dose\cdot F}{CL}$$

2 $${AUC}_{0-inf,lung}= \frac{{Dose}_{inhaled}\cdot {F}_{Pul}}{{k}_{a} \cdot {V}_{lung}}$$

V_Lung_ was set to 0.840 L based on literature values for lung weight [21]. A more detailed description of the NCA can be found in the supplementary material (Supplementary Material, S4). The above-mentioned metrics were calculated separately for each individual. Mean values were used for comparison to model-predicted population values.

### Evaluation criteria

#### Evaluation of the structural identifiability of pulmonary absorption models

Pulmonary absorption models were deemed equivalent with regard to the systemic exposure if the newly predicted plasma concentration–time profiles deviated from the originally simulated PK profiles by less than five percent, based on the geometric mean fold error (GMFE) comparing both profiles [26]. The GMFE was considered the best metric for this comparison, as it simultaneously compares the full plasma concentration–time profiles and equally weights under- and overpredicted concentrations:

3 $$GMFE = 10^{{\frac{{\sum {\left| {log_{{10}} \left( {\frac{{Pred_{i} }}{{Obs_{i} }}} \right)} \right|} }}{N}}}$$

with *Obs*_*i*_ denoting the *i*th plasma concentration simulated by the original Simulation Model, and *Pred*_*i*_ being the *i*th plasma concentration predicted by the Estimation Model. N denotes the total number of simulated data points.

Two different pulmonary exposure metrics were considered to determine the overall pulmonary exposure (area under the lung concentration–time curve, AUC_0-inf,lung_) and the retention in the lungs (time to reach the before simulated pulmonary concentration after 24 h, t_C24h,lung_). The t_C24h,lung_ was considered to evaluate the duration of exposure instead of the more common terminal (pulmonary) elimination half-life, due to the fact that the terminal pulmonary half-life would be mainly dependent on the slowest absorption rate, whereas t_C24h,lung_ is a compromise by all (up to three) parallel pulmonary absorption processes. Furthermore, we are not aware of any inhaled drugs, for which the dosing interval is longer than 24 h so that we consider t_C24h,lung_ the better surrogate for this analysis than the terminal half-life. Adequate inference of lung exposure was considered for both metrics if the reevaluated value was within 80–125% of the originally simulated values, analogous to commonly applied bioequivalence criteria [27].

#### Performance of pulmonary absorption models in a clinical trial setting

For the population PK analyses, the acceptance criterion was 80–125% for AUC_0-inf,plasma_. As the pulmonary PK metrics were inferred rather than measured, the related predictions of the pulmonary exposure were considered acceptable if predictions were within twofold of the true value for both the extent and duration of pulmonary exposure.

The accuracy of the exposure metrics was further evaluated based on the respective distribution (median, 2.5th and 97.5th percentiles of the predicted metrics). Furthermore, the relative bias of the mean (%Bias) was evaluated as follows, inserting the newly predicted and originally simulated exposure metrics as *Pred* and *Obs*, respectively, and the total number of predicted values as *N*:

4 $$\%Bias= \frac{1}{N} \cdot \sum \frac{(Pred-Obs)}{Obs} \cdot 100$$

## Results

### Evaluation of the structural identifiability of pulmonary absorption models

**Fig. 3:**
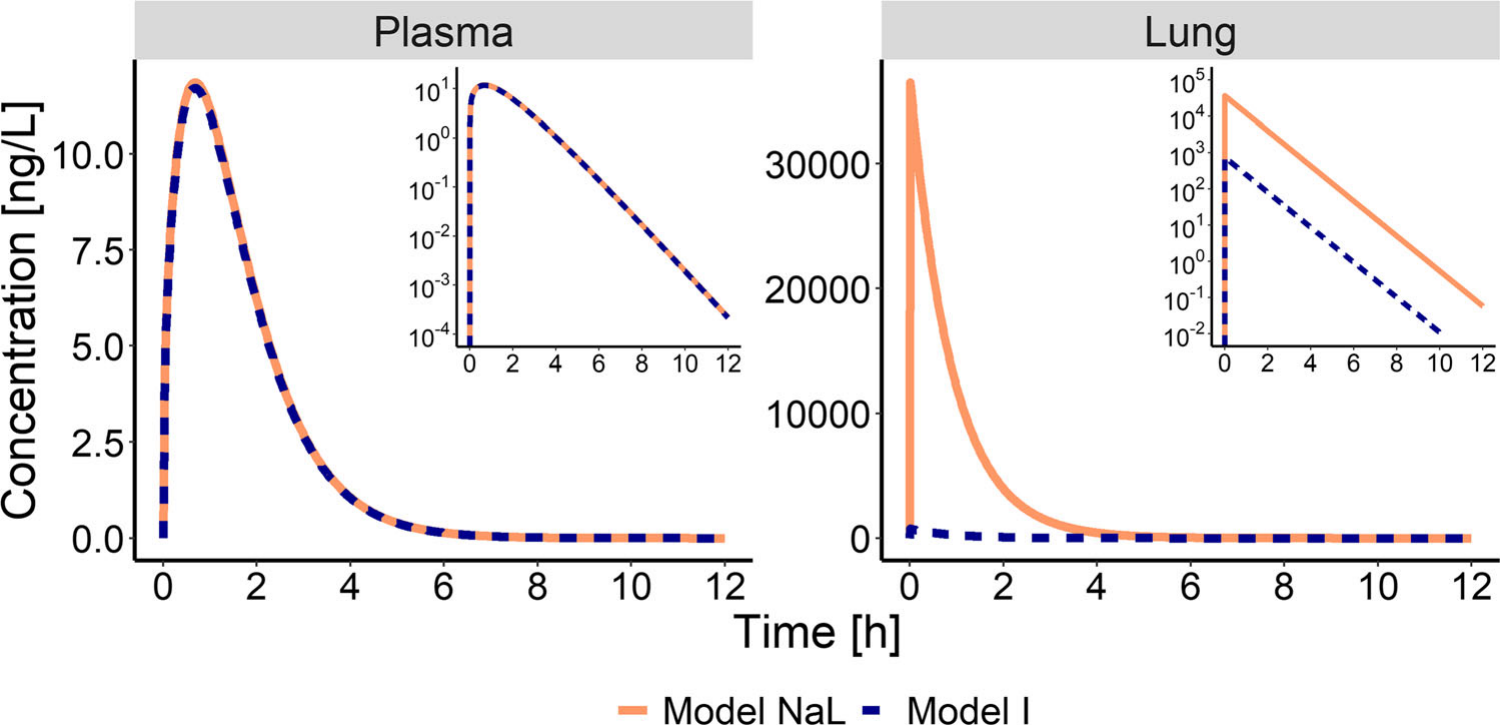
Simulated plasma (left) and lung (right) concentration–time profiles. Solid lines: ‘Model NaL’ used for simulation. Dashed lines: Predictions based on ‘Model I’ used for re-estimation

The results of the evaluation of structural identifiability can be grouped into four different scenarios regarding the predefined criteria (deviation of plasma profiles by less than 5%, and pulmonary AUC_0-inf,lung_ and t_C24h,lung_ within 80–125% of the simulated values), as shown in Table 2. Scenario (1) both plasma and lung exposure were described adequately; Scenario (2) plasma exposure was described adequately, but pulmonary exposure was not; Scenario (3) plasma concentration–time profiles were not captured well; and Scenario (4) the parameters were not identifiable (model not structurally identifiable). Only scenario 2 would result in inferring wrong pulmonary exposure without the possibility to discriminate the models based on plasma concentration data. One example for scenario 2 is the simulation with ‘Model NaL’ and re-estimation with ‘Model I’, as shown in Fig. 3. In this case, choosing the ‘wrong’ pulmonary absorption model would result in a 49.0-fold error in pulmonary AUC. Even though this might be expected, the analyses still underlined that these models can theoretically well describe clinical plasma PK data but would result in completely different pulmonary PK profiles (compare Fig. 3).

**Table 2:**
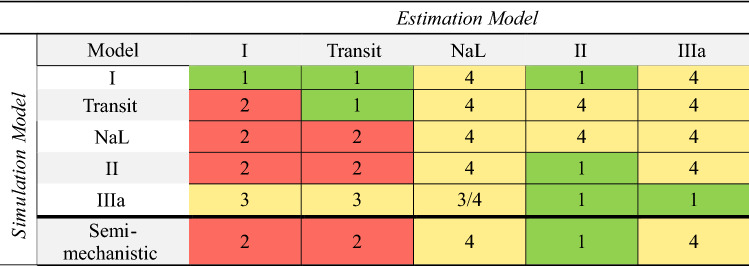
Description of systemic and pulmonary exposure

Rows: simulation model, columns: estimation model. 1: Adequate description of plasma and lung exposure; 2: adequate description of plasma, inadequate description of pulmonary exposure; 3: inadequate description of plasma PK (lung PK not investigated); 4: non-identifiable parameters. The color coding denotes the severity of error in inferring on pulmonary PK by choosing the Estimation Model over the true model: green: No relevant error, yellow: theoretically relevant error, but distinction based on plasma PK data possible; red: relevant error, no distinction possible based on plasma PK data (Color figure online)

Examples for Scenarios 1 and 3 can be found in the supplementary material (Supplementary Material S3, Fig. S3). Plasma PK was described well in most simulation-estimation evaluations, except for simulations with ‘Model IIIa’. As expected, non-identifiable parameters were generally encountered when trying to fit more complex models (with more parameters) to data generated with simpler models, e.g. estimation with ‘Model II’ and ‘IIIa’ on simulated data of models ‘I’, ‘Transit’, and ‘NaL’. ‘Model NaL’ was unidentifiable if used for re-estimation, due to the correlation between F_Pul_ and k_NaL_.

### Link between empiric and mechanistic PK modeling

Of all models, only ‘Model II’ allowed adequately inferring on pulmonary exposure that was simulated with the semi-mechanistic model. The omission of redistribution of drug from plasma to the lung did not impact on inferred pulmonary exposure, showing a deviation from the simulation of < 1% for both extent and duration of pulmonary exposure (Supplementary Material S2.3, Fig. S2).

### Performance of pulmonary absorption models in a clinical trial setting

#### Simulation with ‘Model II’

**Fig. 4:**
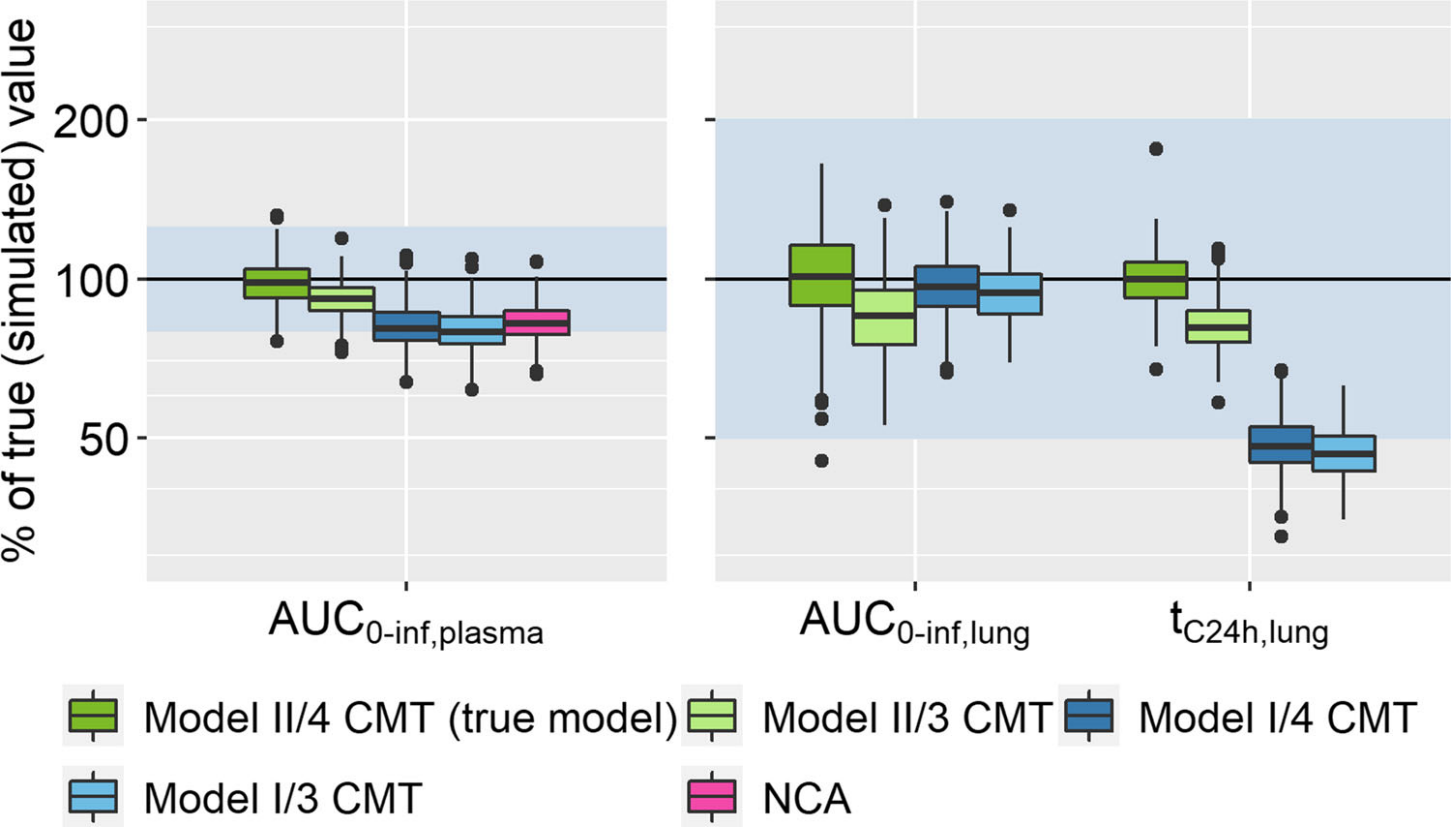
Exposure metrics estimated on data simulated with ‘Model II’/4cmt using the PPP method. 3 CMT and 4 CMT denote the number of systemic PK compartments; ‘Model I’ or ‘II’ describes the pulmonary absorption model, with one or two parallel absorption processes, respectively. The shaded area represents the accepted range (80–125% for plasma, twofold deviation for lung metrics). Number of successful estimations: 424 (II/4 CMT), 361 (II/3 CMT), 464 (I/4 CMT), and 471 (I/3 CMT)

Most of the Estimation Models used in the population simulation-estimation study involving ‘Model II’, with the exception of estimation with ‘Model I’ using the PPP method, were able to describe both plasma and pulmonary AUC_0-inf_ adequately, with over 90% of the runs within 80–125% of the true value. However, when comparing the retention in the lung, as determined by t_C24h,lung_, both Estimation Models including ‘Model I’ for pulmonary absorption deviated substantially from the true value with a bias of -60% to -50% (see Table 3). Applying the correct model resulted in all but one of the evaluations (using the PPP method) within twofold of the true AUC_0-inf,lung_. For the t_C24h,lung_, 100% %were within twofold. In all cases, the true model (four compartment systemic model and/or ‘Model II’) performed best with regard to the AIC. When using the PPP method, the estimates returned by the true model were slightly less precise in comparison to the other model structures with a median and 2.5th and 97.5th percentiles of 101% (62.8%, 147%) of the true value for AUC_0-inf,lung_ (see Table 3). Figure 4 shows an exemplary distribution of these exposure metrics.

**Table 3:** Median [2.5th and 97.5th percentile], and width of the 95% interval of PK metrics inferred for oral drug inhalation

Absorption model	Systemic model	AUC_0-inf,plasma_			AUC_0-inf,lung_ (extent of pulmonary exposure)			t_C24h,lung_ (duration of pulmonary exposure)		
		PPP	IPP	ALL	PPP	IPP	ALL	PPP	IPP	ALL
Model II	4 CMT	98.5% [83.1%, 118%], 34.9%	99.5% [85.7%, 116%], 30.3%	98.6% [84.7%, 116%], 31.3%	101% [62.8%, 147%], 84.2%	102% [74.1%, 134%], 59.9%	103% [76.5%, 135%], 58.1%	24.0 h [19.2 h, 29.4 h], 10.2 h	23.9 h [19.0 h, 29.0 h], 10.0 h	23.8 h [19.1 h, 28.8 h], 9.69 h
Model II	3 CMT	91.9% [76.6%, 107%], 30.4%	97.3% [83.3%, 113%], 29.3%	96.6% [82.5%, 111%], 28.5%	85.1% [60.3%, 118%], 57.7%	82.4% [55.5%, 117%], 61.5%	79.3% [59.3%, 107%], 47.7%	19.6 h [16.0 h, 24.1 h], 8.07 h	19.9 h [15.7 h, 25.9 h], 10.2 h	18.9 h [16.0 h, 22.4 h], 6.41 h
Model I	4 CMT	80.7% [68.3%, 97.1%], 28.8%	94.4% [81.0%, 111%], 30.0%	93.6% [80.4%, 110%], 29.6%	96.5% [74.0%, 128%], 54.0%	96.0% [74.1%, 119%], 42.5%	90.6% [68.4%, 118%], 49.2%	11.7 h [9.20 h, 14.7 h], 5.50 h	9.88 h [7.50 h, 12.5 h], 5.00 h	9.46 h [7.15 h, 12.3 h], 5.19 h
Model I	3 CMT	79.2% [67.2%, 94.6%], 27.4%	91.2% [78.4%, 107%], 28.6%	90.1% [77.2%, 105%], 27.8%	94.1% [75.1%, 118%], 42.9%	91.4% [72.0%, 115%], 43.0%	91.0% [74.2%, 114%], 40.2%	11.3 h [9.10 h, 13.9 h], 4.80 h	9.52 h [7.50 h, 12.1 h], 4.60 h	9.62 h [7.62 h, 12.1 h], 4.48 h

Values for AUCs are given as percentage of the true value, t_C24h,lung_ is given as absolute values. Simulation Model: ‘Model II/4CMT’. CMT: compartments. PPP: fixed systemic population parameters; IPP fixed individual parameters; ALL: simultaneous fit of all parameters

Choosing a three compartmental systemic disposition model instead of four compartments resulted in only slightly worse predictions in this analysis. However, the combination of a three-compartment systemic model with ‘Model II’ for pulmonary absorption converged in only 50–70% of runs vs. convergence of > 85% in the other scenarios. In terms of stability, the PPP method performed best, while the ALL method was slightly more unstable than the IPP approach.

Regarding the modeling approaches, PPP, IPP, and ALL performed comparable with regard to predicted systemic but also inferred extent and duration of pulmonary exposure (see Table 3). One notable exception was the combination of the PPP approach with ‘Model I’ as the Estimation Model. In this case, AUC_0-inf,plasma_ after inhalation was less often predicted well (only 55% and 45% of successful runs within 80% to 125% of the true value, for a three and four compartmental systemic PK model, respectively). However, the prediction of t_C24h,lung_ was marginally better than with the other two approaches (28.7% and 40.5% of predictions within twofold of the true value, compared to 3–5% with the other methods). A comparison of the precision and the parameter estimates acquired using the three methods for the true model can be found in the supplementary material (Supplementary Material S6, Table S5). Model predictions were also compared to the results from the NCA. While the AUC_0-inf,plasma_ was described adequately for most evaluations, calculation of the MAT resulted in negative values for some subjects, preventing the calculation of AUC_0-inf,lung_. Melin et al. [15] also encountered this in the original publication.

#### Simulation with ‘Model IIIa’

**Fig. 5:**
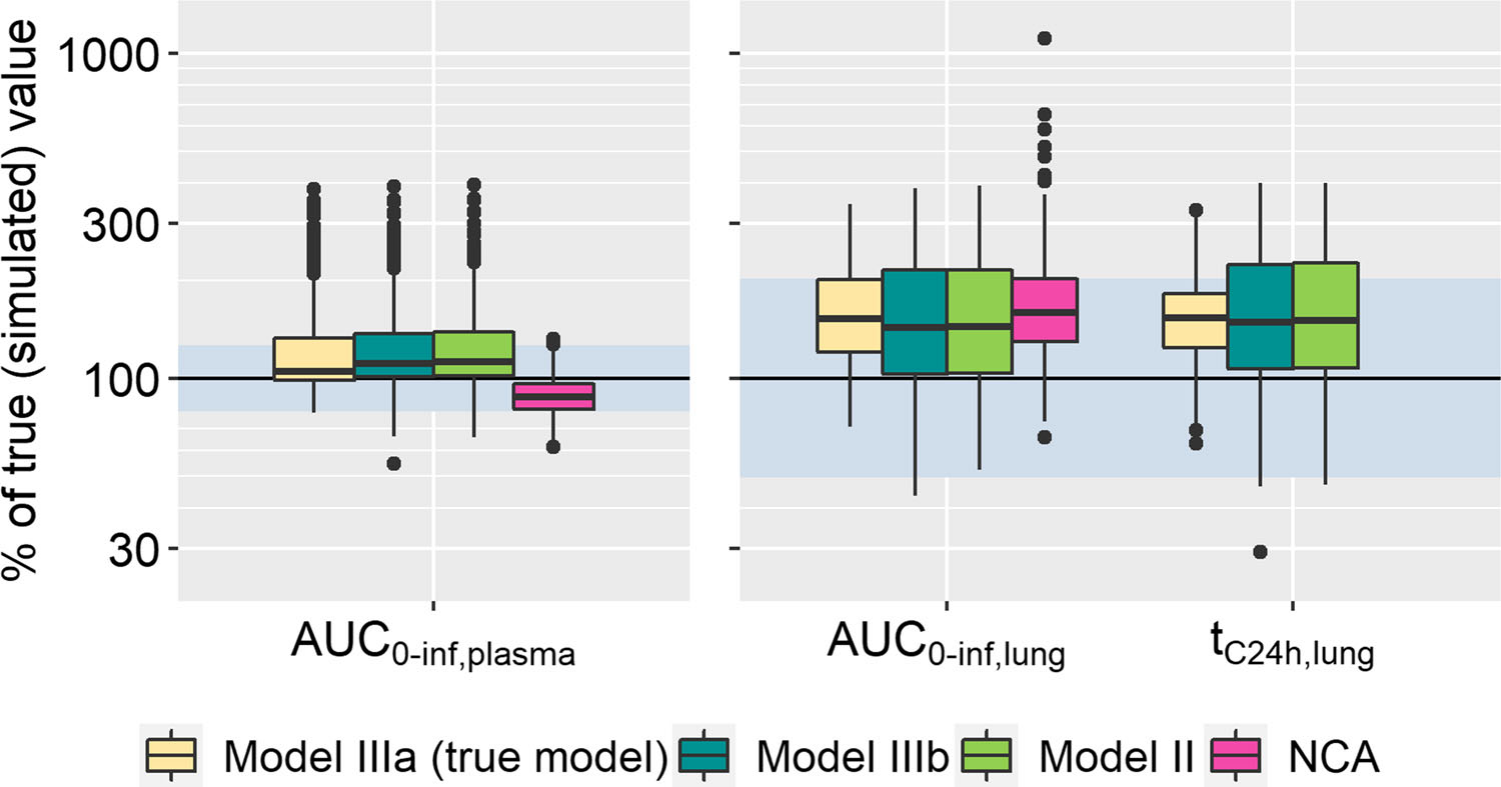
Exposure metrics estimated on data simulated with ‘Model IIIa’. The shaded area represents the accepted range (80–125% for plasma, twofold deviation for lung metrics). Number of successful estimations: 473 (IIIa), 472 (IIIb), and 474 (II)

The simulation/re-estimation analysis with ‘Model IIIa’ as the Simulation Model resulted in a systematic overestimation of lung exposure, regardless which model was used for estimation (Fig. 5). AUC_0-inf,plasma_ was mostly estimated well. In this case too, the true model (‘Model IIIa’) was superior with regard to the AIC. All model predictions tended towards overestimation (bias of 23.4%, 26.4%, and 27.8% for ‘Model IIIa’, ‘IIIb’, and ‘II’, respectively). ‘Model IIIa’ gave overall more precise but slightly biased estimates, 105% (87.4%, 253%) of the original value (median, 2.5th and 97.5th percentiles) for AUC_0-inf,plasma_, 153% (89.0%, 299%) for AUC_0-inf,lung_, and 38.1 h (22.4 h, 65.0 h) for t_C24h,lung_. 74.8%, 97.9%, and 100% of the evaluations were within twofold, threefold, and fivefold of the true AUC_0-inf,lung_. For the t_C24h,lung_ 82.4%, 99.2%, 100% were within twofold, threefold, and fivefold, respectively. In comparison, the estimates by the other two models were less precise. The respective median values with 2.5th to 97.5th percentiles for all Estimation Models can be found in Table 4.

**Table 4:** Median [2.5th and 97.5th percentile], width of the 95% interval, and relative bias of the mean (%Bias) for the population analysis based on datasets simulated with ‘Model IIIa’

	Absorption model	AUC_0-inf,lung_ (extent of pulmonary exposure)					t_C24h,lung_ (duration of pulmonary exposure)		
		Total	Inadequate systemic PK			Adequate systemic PK	Total	Inadequate systemic PK	Adequate systemic PK
Median [2.5th, 97.5th percentiles], width of the 95% interval	Model IIIa	153% [89.0%, 299%], 210%	162% [100%, 299%], 199%			105% [81.5%, 120%], 38.5%	38.1 h [20.0 h, 64.5 h], 44.5 h	38.1 h [22.4 h, 65.0 h], 42.6 h	24.7 h [18.7 h, 29.6 h], 10.9 h
	Model IIIb	144% [69.3%, 337%], 268%	153% [68.1%, 340%], 271%			90.2% [71.1%, 107%], 35.9%	41.4 h [15.1 h, 84.8 h], 69.6 h	38.4 h [14.9 h, 85.2 h], 70.2 h	21.8 h [16.6 h, 26.8 h], 10.3 h
	Model II	145% [70.1%, 341%], 271%	155% [69.2%, 345%], 276%			91.2% [71.6%, 108%], 36.4%	41.8 h [15.6 h, 85.5 h], 69.8 h	38.8 h [15.3 h, 86.3 h], 70.9 h	22.1 h [16.8 h, 27.1 h], 10.3 h
	NCA	160% [92.8%, 339%], 246%	176% [110%, 338%], 228%			158% [89.0%, 336%], 247%	–	–	–
%Bias (mean)	Model IIIa	67.0%	76.7%			2.88%	58.6%	67.1%	2.86%
	Model IIIb	66.2%	77.9%			− 11.0%	72.7%	86.6%	− 7.89%
	Model II	67.7%	79.5%			− 10.2%	74.2%	85.1%	− 8.99%
	NCA	126%	124%			139%	–	–	–

**Fig. 6:**
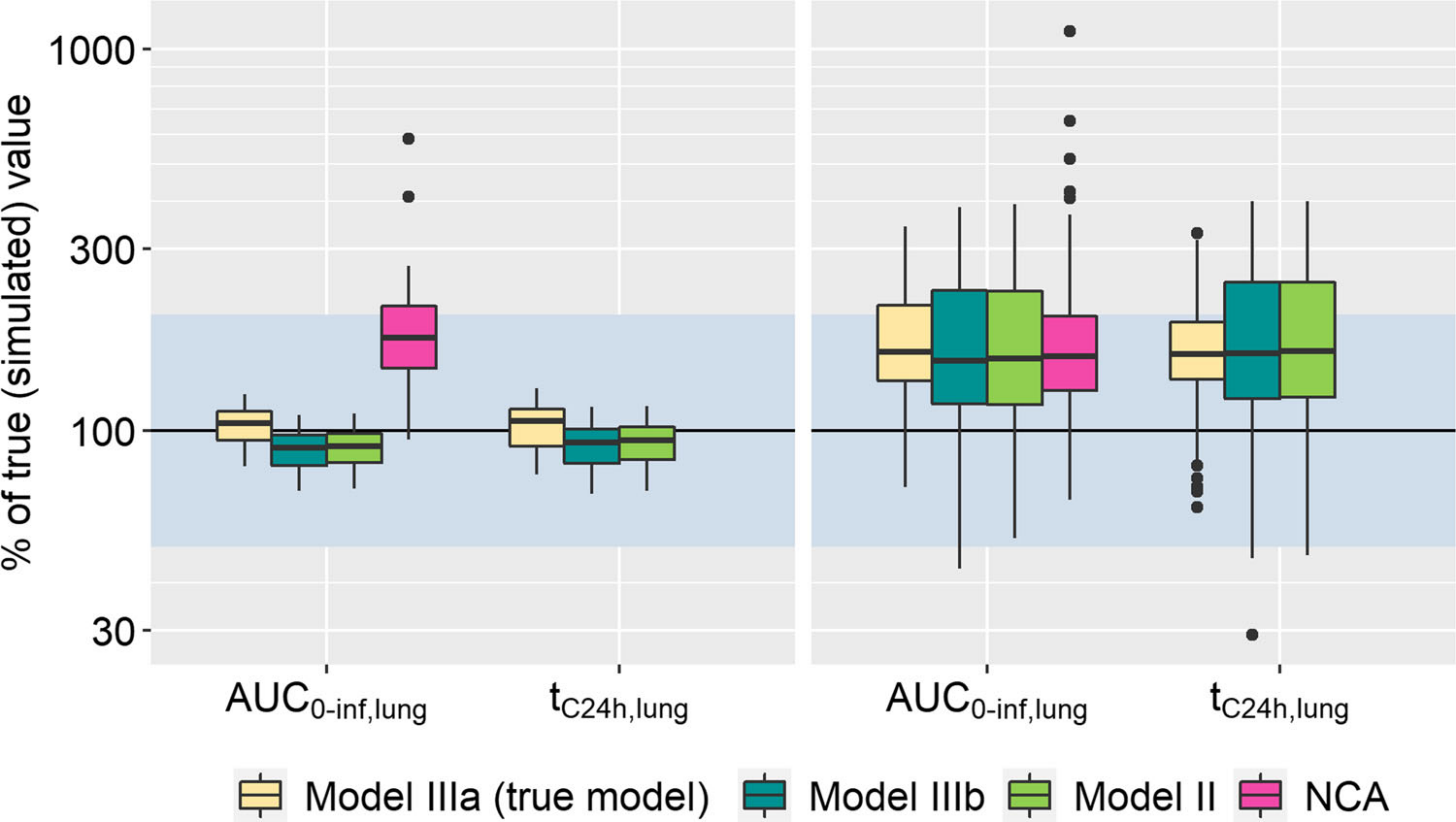
Exposure metrics estimated on data simulated with ‘Model IIIa’ separated by the adequacy of systemic PK parameters (deep compartment). Left panel: adequate systemic PK; right panel: inadequate systemic PK. Adequate systemic PK: Parameters Q2 and V2 within 80–125% of the true values and the corresponding NCA predictions. The shaded area represents the accepted range (80–125% for plasma, twofold deviation for lung metrics). Number of estimations with adequate systemic PK: 58 (each of the models). Number of estimations with inadequate systemic PK: 415, 414, and 416 for IIIa, IIIb, and II, respectively

To further investigate potential reasons for the overestimation and imprecision of lung exposure, the estimates of the systemic PK parameters were further investigated. The parameter estimates characterizing the distribution to the deep tissue compartment (Q2 and V2) showed high variability, with V2 ranging from 10 to 2000% of the true value used for data simulation. Further investigations revealed a correlation between V2 and the pulmonary absorption rates. As a follow-up, AUC_0-inf,lung_ and t_C24h,lung_ were compared between runs with accurate parameters (Q2 and V2 within 80–125% of the true values) and those with inaccurate parameters. The resulting distributions can be seen in Fig. 6. The runs with Q2 and V2 estimates close to their true values showed no overestimation of pulmonary exposure; all predictions were within twofold and over 75% of runs within 80–125% for both pulmonary exposure metrics. Precision of the predictions also improved greatly (median and 2.5th and 97.5th percentiles: 105% (81.5%, 120%) of the true value for AUC_0-inf,lung_; 24.7 h (18.7 h, 29.6 h) for t_C24h,lung_, ‘Model IIIa’). While the difference between the models was marginal, ‘Model IIIa’ resulted in the best predictions (Table 4). Given the published clinical designs for the i.v. study, the systemic disposition parameters could only be adequately estimated in 11.6% of the simulation estimation studies. The majority of runs with inaccurate systemic PK parameters presented a substantial overestimation of both AUC_0-inf,lung_ and t_C24h,lung_ with approximately 30% showing a deviation of greater than twofold from the original.

## Discussion

It is challenging to evaluate the local pharmacokinetics after drug administration, especially when the target organ is identical to the site of administration. However, for many locally administered drugs it is assumed that local drug concentrations provide efficacy [28]. In these cases, a good understanding of the local PK is desirable. PK modeling based on plasma PK data might be one of the easiest approaches to infer pulmonary exposure after drug inhalation. In contrast to experimental determination of pulmonary exposure, modeling does not require additional invasive exposure measurements or imaging data. Therefore, the aim of this work was to evaluate the possibilities and limitations of using empirical PK models for pulmonary absorption to infer both the extent and duration of pulmonary PK. This investigation showed that empirical PK modeling can be a valuable tool to infer pulmonary PK. Finally, based on the results a strategy for PK (modeling) analyses was developed, including (1) the right choice of pulmonary absorption models, and (2) a quantitative evaluation of bias and precision of inferring the extent and duration of pulmonary exposure based on realistic clinical datasets.

As a first step of performing a modeling analysis to infer the pulmonary PK, suitable model structures should be selected based on *prior* knowledge about relevant pulmonary PK processes. The reason is that most of the here investigated absorption models were discussed to have a physiological interpretation, ranging from non-absorptive loss via mucociliary clearance or pulmonary metabolism [16] to parallel absorption processes in different lung regions [9]. The only investigated model without an obvious underlying physiological reasoning is the ‘Model Transit’, as drug absorption can start everywhere in the lung simultaneously (e.g., conducting airways and alveolar space). Therefore, and as this empiric transit absorption model is rarely applied to characterize pulmonary absorption, this model structure will not be further discussed.

Pre-selection of plausible models can be done for example based on in vitro experiments (e.g. dissolution measurements [29] and/or metabolic stability in lung slices [30]), or preclinical in vivo studies. Without this data, this modeling analysis showed that no inference on pulmonary PK is possible (i.e., different models describing the plasma PK adequately resulted in approximately 50-fold differences with regard to pulmonary exposure). If prior knowledge suggests that pulmonary metabolism is present or that mucociliary clearance is relevant due to slow dissolution, a model-based approach with implementation of these processes is necessary to achieve adequate predictions of pulmonary exposure (e.g., ‘Model NaL’). It has to be noted that, even when selecting the right model for a drug with non-absorptive loss, the parameter estimation process resulted in unidentifiable parameters. Sakagami et al. suggested that this instability can be circumvented by fixing the lung dose [31]. However, this requires detailed information about the lung dose, which is subject to great variability, both between subjects and between occasions [9, 32, 33]. It is therefore debatable, if empirical PK analysis based on plasma data will provide valuable insights into pulmonary PK for this scenario.

If the relevance of pulmonary metabolism and mucociliary clearance is negligible, it is possible to explore pulmonary PK by implementation of parallel absorption processes (‘Model I’–‘III’). The structural identifiability evaluation showed, that in one case (simulation with ‘Model II’ and re-estimation with ‘Model I’), the pulmonary absorption models could not be distinguished based on plasma PK data, according to the predefined criteria, yet yielded different outcomes for pulmonary exposure. While both model candidates provided adequate predictions of systemic PK and the extent of pulmonary exposure, the duration of lung retention metric t_C24h-lung_ was significantly underestimated with the less complex ‘Model I’. This might have consequences for selecting dosing schemes when the dosing intervals are pre-selected based on PK rather than PD readouts.

**Fig. 7:**
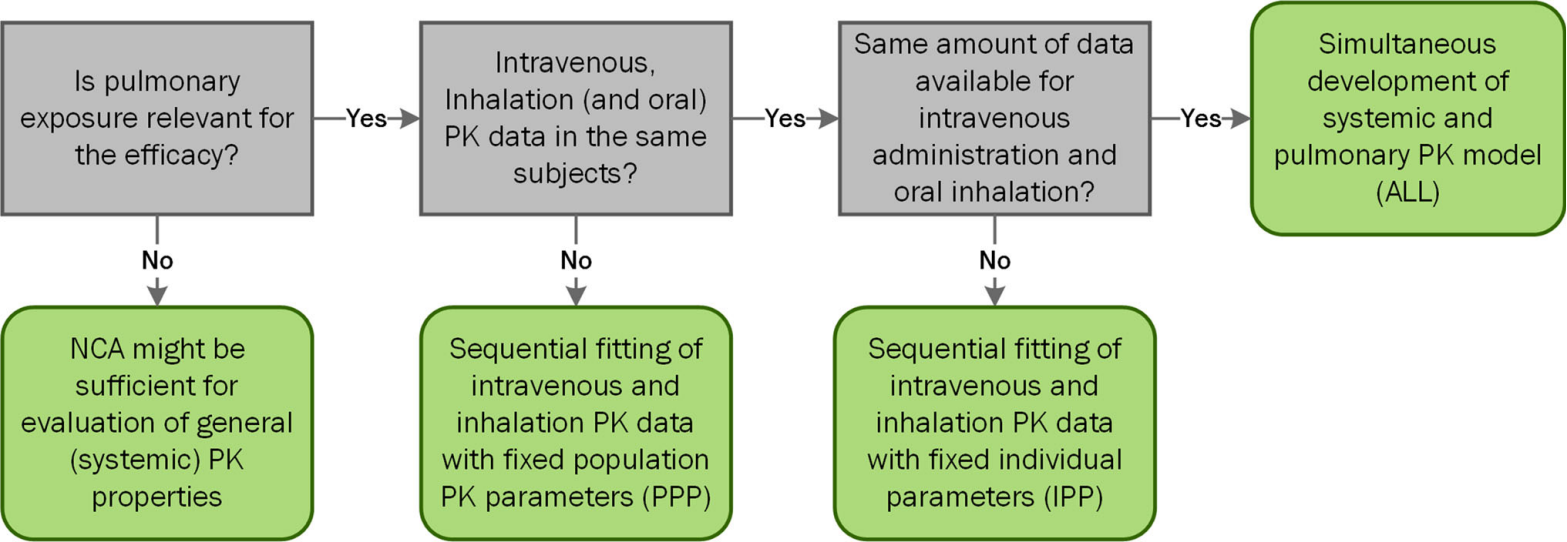
Suggested decision tree for choosing a modeling approach

While the first part of our study was based on full PK profiles without any simulated variability to evaluate the structural identifiability and inter-changeability of the models, clinical data is typically analyzed with a population (PK) approach to quantify different variability components (inter-individual, intra-individual, inter-occasion, etc.). Both inhalation and i.v. data are required to perform deconvolutions. Unfortunately, i.v. data is rarely available in the same individuals as inhalation data. Therefore, an understanding of the implications is required, and a strategy has to be developed, how to best perform such a population approach. To this end, it is helpful to have an overview about the opportunities and limitations of the available options. The PPP method is the most widely applicable method and can always be applied if i.v. and inhalation data are present. Both IPP and ALL were found to be reasonable methods if i.v. and inhalation PK have been measured in the same subjects. However, in light of the marginal differences regarding parameter estimates (Supplementary Material, Table S5) and estimated PK metrics in this investigation, it is debatable if the added effort of conducting i.v. and inhalation trials in the same subjects is justified. A decision tree showing the requirements for each approach is shown in Fig. 7.

In general, modelling provided fairly accurate predictions for extent and duration of pulmonary exposure (most predictions within twofold of the true value), given that the correct model structure can be identified. Misspecification of the absorption model could result in failure to capture the duration of exposure, as could be seen in the analysis based on ‘Model II’. Analogous to the structural identifiability analysis, re-evaluation of pulmonary exposure metrics based on simulated clinical datasets with only one absorption process adequately predicted the pulmonary AUC, but substantially underestimated the retention (only 3% to 40% within twofold of the true value, i.e. t_C24h,lung_ < 12 h). Based on these PK estimates alone, bi-daily dosing might be chosen instead of the ‘true’ once-daily administration, showing that PD readouts should always be considered, when possible for the respective mechanism of action, to make these decisions. However, the true model would always have been chosen based on statistical model-selection criteria. Therefore, when carefully performing such a modeling analysis, a proper discrimination between these models seems possible. Finally, based on the here presented findings, a post-hoc analysis similar to the presented approach based on the finally selected model structure, the estimated PK parameters, and the investigated data set should be considered to assess the robustness of the model-based inference on pulmonary PK. If the results prove to be reliable and do not contradict other available information, the predictions should provide a reasonable basis to support dosing and posology decisions.

It should be kept in mind that even with the ‘correct’ pulmonary absorption model and an adequate modeling strategy there are still some critical aspects to consider. For example, the population PK analysis based on ‘Model IIIa’ demonstrated a bias in both pulmonary exposure metrics caused by inaccurate estimation of systemic PK parameters. Here, a high correlation was found between the volume of distribution of the systemic deep tissue compartment V2 and the slow pulmonary absorption rate. Probably, the slow absorption rate constant (k_slow_) was compensating for underestimation of the systemic terminal half-life based on i.v. data. Lower estimates for V_2_ led to a shorter terminal elimination half-life, and in the inhalation trials with longer sampling times (up to 8 days after the last dose), the terminal half-life of ~ 30 h was therefore attributed to the slow absorption from the lung. Even in the original publication, the absence of flip-flop kinetics could only be demonstrated by the inclusion of urine data [9]. This potential bias in both extent and duration of pulmonary exposure further underlines the importance of high quality i.v. PK data and could have possibly been avoided by extending the sampling times after i.v. administration. However, this may not always be feasible, as orally inhaled drugs potentially produce (dose-limiting) side effects precluding the use of higher doses to be able to observe the ‘true’ terminal phase in the PK profile [34, 35]. The identification of flip-flop kinetics for an inhaled drug with a high volume of distribution may indicate that the terminal phase after i.v. administration is not adequately captured by the PK data. This is even more likely if the long pulmonary retention is hypothesized to be the result of high tissue affinity to the lung, as this should be relevant after both i.v. administration and inhalation. In these cases, the slowest absorption constant should not be smaller than the elimination constant. Interestingly, for’Model IIIa’, only ~ 10% of the simulation-estimation analyses allowed adequately estimating the systemic disposition parameters. This also means that in ~ 90% of the analyses there would have been a relevant risk of overestimating the extent and duration of pulmonary exposure. However, even in these cases, given the correct model structure, most predictions (> 70%) were still within twofold and less than 3% outside threefold of the true value.

Last but not least, the population PK modeling approaches were also compared to the commonly applied NCA, inferring on pulmonary retention based on the NCA-based pulmonary absorption rate. These analyses, performed in parallel to the population PK analyses, yielded ambivalent results for both scenarios. While the NCA performed on the dataset simulated with ‘Model IIIa’ resulted in plausible, yet biased values, the simulation with ‘Model II’ could not be analyzed with an NCA, as for some individuals the MRT after inhalation was shorter than after i.v. administration. Even for individuals with a positive MAT, the mean predicted AUC_0-inf,lung_ was over tenfold higher than the true value (a more detailed investigation of this can be found in the online Supplementary Material). Therefore, based on our analysis, an NCA-based deconvolution of the data cannot be recommended to infer pulmonary exposure.

Few limitations of the present study are acknowledged: One limitation of all the here investigated model structures is that re-distribution from plasma to lung tissue is not accounted for, potentially leading to underestimation of lung concentrations at later time points. However, the comparison of the investigated models with a semi-mechanistic model for salmeterol that does include backflow to the lung showed only minimal deviations in the predicted lung tissue. This suggests that the impact on inferred lung exposure was negligible in this investigation. It should be considered that salmeterol displays high systemic clearance after drug absorption from the lung. For drugs with slower elimination from the systemic perfusion, also the relevance of re-distribution to the lungs would increase.

Another limitation is that investigated models cannot discriminate between dissolution and absorption. This may not be a problem for some of the investigated drugs (e.g. olodaterol for ‘Model IIIa’), which are dissolving very quickly (or are already administered as a solution). However, the absorption of fluticasone propionate (‘Model I’) has been postulated to be limited by its dissolution rate, possibly even masking parallel absorption of dissolved drug with differing rates limiting the applicability of the presented approach. Furthermore, as pulmonary concentrations of inhaled drugs can differ regionally due to local physiology and deposition patterns, considering the averaged drug concentrations of the whole lung might not provide entirely accurate depictions of actual target site concentration [1, 36]. In some cases, even making the best use of the plasma PK data, plasma concentrations may not be a good surrogate, e.g. due to accumulation in lung tissue (active transport, lysosomal trapping). Here, additional information about the relevant processes may help the interpretation of results.

A limitation of sequentially fitting i.v. and inhalation data from different individuals (PPP approach) is the assumption that the systemic PK of subjects is comparable in the i.v. and the inhalation PK studies. This might not always hold true. For example, it might be important to consider if the i.v. study was performed in healthy volunteers, and inhalation trials included in the dataset were conducted in patients with potentially altered physiology. In general, sequentially fitting the i.v. and inhalation data (PPP and IPP) might result in underestimation of the parameter uncertainty for the pulmonary absorption parameters [23]. The influence of sequential fitting methods on parameter uncertainty was previously investigated in more detail by Zhang et al. [37, 38]. Notably, the PK analyses in this work were performed with only one set of simulation parameters per pulmonary absorption model, directly based on the original publications to ensure that the tested scenarios and study designs are realistic. A repetition with different simulation parameters may result in different conclusions. For example, the lower the difference between the parallel absorption rate constants, the harder it might be to differentiate between different pulmonary absorption processes.

Moreover, the analyses did not account for concentrations below the lower limit of quantification, which can have great impact on reasonable study designs and result in distortion of parameter estimates [39] but was beyond the scope of the present study. The impact of data below the limit of quantification was investigated and described in the original publications of the here chosen examples. Neither Borghardt et al. [9] nor Melin et al. [15] reported a significant effect of accounting for missing data in the modeling process. Due to the previous investigations and conclusions for the model drugs, we decided to not include these characteristics in our evaluations. However, it cannot be precluded that unaccounted-for missing data may lead to false conclusions in other cases, especially as inhaled doses are typically low in the µg range and this can result in high fractions of data being below the limit of quantification.

The analysis based on clinical datasets included only proportional residual variability, which might influence parameter estimation, as this may not adequately represent the measurement errors at lower concentrations. Investigating the impact of identifying the correct residual variability model on inferring pulmonary exposure may be an interesting follow-up study.

Even though the here evaluated models are based on physiological reasoning, all of them represent empirical modeling approaches. Until today, the link between these empiric model structures and mechanistic PK models is not systematically established. Adequate implementation of all the relevant pulmonary PK processes after inhalation would require more mechanistic PK models (compare mechanistic PK models e.g. by Boger et al. [40] or Hartung and Borghardt [41]). However, while these more mechanistic PK models would allow simulation of time-resolved PK profiles in different areas of the lung, these mechanistic PK models can typically not be estimated based on available clinical data. In the future, more integrative PK modeling approaches relying on plasma PK data, preclinical in vitro*,* and preclinical in vivo experiments can be expected to allow even better inference on pulmonary exposure and retention times, when mucociliary clearance and slow dissolution kinetics are of relevance. In any case, checking the plausibility of parameter estimates based on prior knowledge is always advisable.

## Conclusion

This work illustrated the value of PK modeling to infer the extent and duration of pulmonary exposure from systemic concentration–time profiles. When the aim is to learn about the pulmonary fate of orally inhaled drugs, our analysis indicated that PK modeling is superior to NCA. It was demonstrated that when selecting the right structural systemic and pulmonary absorption model, which was not always trivial even based on rich clinical datasets, the error in the majority of predictions of extent and duration of pulmonary exposure was less than twofold. Sequential versus simultaneous estimation of systemic and pulmonary PK parameters both provided good results and only showed marginal differences in the prediction of pulmonary PK. It was also demonstrated that inferring the extent of pulmonary exposure was more robust in comparison to inferring the pulmonary retention if the wrong structural absorption model was used. However, even with very rich clinical datasets, still a moderate risk remains that the pulmonary retention is not adequately inferred. Therefore, while modelling was proven to be a useful tool to learn about the pulmonary fate of inhaled drugs, care should be taken when basing decisions about doses and especially dosing posology solely on inference from plasma PK. Given the uncertainties, a post-hoc simulation-estimation analysis to evaluate the robustness of model predictions would be recommended, and if possible, model-based predictions of the pulmonary PK should always be used in conjunction with available PD data.

## Supplementary Information

Below is the link to the electronic supplementary material.

Supplementary file1 (PDF 685 kb)
