# Supplementary material for: Inferring pulmonary exposure based on clinical PK data: accuracy and precision of model-based deconvolution methods
Source: J Pharmacokinet Pharmacodyn. 2021 Sep 28;49(2):135–49. doi: 10.1007/s10928-021-09780-x (PMC8940815; doi:10.1007/s10928-021-09780-x)
Supplement: Supplementary file 1 — Supplementary file1 (PDF 685 kb) [file 10928_2021_9780_MOESM1_ESM.pdf]

Supplementary Material

Table of Contents

S1. Abbreviations.. 1

S2. Model Parameterization.. 2

S2.1. Ordinary differential equations.. 2

Model_IIIa (Olodaterol, Borghardt et al. (1)).. 2

Model_II (AZD5423, Melin et al. (2)).. 2

Model_I (Fluticasone propionate, Krishnaswami et al. (3)).. 3

Model_NaL (inhaled insulin, Sakagami et al. (4)).. 3

Model_Transit (PF-00610355, Diderichsen et al. (5)).. 3

S2.2. Parameter values used for simulation.. 4

S2.3. Semi-mechanistic model.. 6

S3. Exemplary Figures: Scenarios 1 and 3.. 7

S4. Non-compartmental analysis.. 8

S6. Comparison of parameter estimates between PPP, IPP, and ALL.. 10

S1. Abbreviations

Table S1. Abbreviations of PK parameters.

CL	Systemic clearance
Vn	Volume of distribution of the nth compartment
Qn	Intercompartmental clearance to the nth compartment
F_{pul}	Pulmonary bioavailablity/Lung deposited dose (Model NaL)
PF1	First proportionality factor
PF2	Second proportionality factor
K_{slow}	Slow pulmonary absorption rate constant
K_{med}	Intermediate pulmonary absorption rate constant
K_{fast}	Fast pulmonary absorption rate constant
K_{NaL}	Non-absorptive loss rate constant
$K_{Transit}$	Transit rate constant
IIV	Interindividual variability
$Prop_{iv}$	Proportional residual variability after intravenous administration
$Prop_{inh}$	Proportional residual variability after inhalation
PPP	Sequential estimation of systemic and absorption PK parameters (based on intravenous PK and inhalation PK, respectively), estimation of absorption parameters on top of the fixed systemic typical population parameters (thetas) and their variance (omega matrix)
IPP	Sequential estimation of systemic and absorption PK parameters (based on intravenous PK and inhalation PK, respectively), estimation of absorption parameters on top of the fixed individual PK parameters (empiric Bayesian estimates, thetas + etas)

ALL	Simultaneous estimation of systemic and absorption PK parameters on a combined dataset of intravenous and inhalation PK data

S2. Model Parameterization

Parameterization of the fractions of the dose absorbed with a slow, intermediate and fast rate constant ($F_{slow/med/fast}$):

$$F_{slow} = nDose \cdot PF1 \cdot F_{Pul} \quad \text{(S1)}$$

$$F_{med} = nDose \cdot PF2 \cdot (1 - PF1) \cdot F_{Pul} \quad \text{(S2)}$$

$$F_{fast} = nDose \cdot (1 - PF2 \cdot (1 - PF1) - PF1) \cdot F_{Pul} \quad \text{(S3)}$$

With *nDose* being the nominal dose, F_{Pul} being the pulmonary bioavailability, and *PF1* and *2* as the proportionality factors (Parameterization from Borghardt et al. (1)).

S2.1. Ordinary differential equations

Model_IIIa (Olodaterol, Borghardt et al. (1))

dCentral = - (CL/V1 · Central) + (Kfast · Abs.fast) + (Kmed · Abs.med) + (Kslow · Abs.slow) – (Q2/V1 · Central) + (Q2/V2 · Peripheral.1) – (Q3/V1 · Central) + (Q3/V3 · Peripheral.2) – (Q4/V1 · Central) + (Q4/V4 · Peripheral.3)

dPeripheral.1 = (Q2/V1 · Central) – (Q2/V2 · Peripheral.1)

dPeripheral.2 = (Q3/V1 · Central) – (Q3/V3 · Peripheral.2)

dPeripheral.3 = (Q4/V1 · Central) – (Q4/V4 · Peripheral.3)

dAbs.fast = - (Kfast · Abs.fast)

dAbs.med = - (Kmed · Abs.med)

dAbs.slow = - (Kslow · Abs.slow)

dElim = (CL/V1 · Central)

Model_II (AZD5423, Melin et al. (2))

dCentral = -(CL/V1 · Central) + (Kfast · Abs.fast) + (Kslow · Abs.slow) - (Q2/V1 · Central) + (Q2/V2 · Peripheral.1) - (Q3/V1 · Central) + (Q3/V3 · Peripheral.2) - (Q4/V1 · Central) + (Q4/V4 · Peripheral.3)

dPeripheral.1 = (Q2/V1 · Central) - (Q2/V2 · Peripheral.1)

dPeripheral.2 = (Q3/V1 · Central) - (Q3/V3 · Peripheral.2)

dPeripheral.3 = (Q4/V1 · Central) - (Q4/V4 · Peripheral.3)

dAbs.fast = - (Kfast · Abs.fast)

dAbs.slow = - (Kslow · Abs.slow)

dElim = (CL/V1 · Central)

Model_I (Fluticasone propionate, Krishnaswami et al. (3))

dCentral = -(CL/V1 · Central) + (Kmed · Abs.med) – (Q2/V1 · Central) + (Q2/V2 · Peripheral.1) – (Q3/V1 · Central) + (Q3/V3 · Peripheral.2) – (Q4/V1 · Central) + (Q4/V4 · Peripheral.3)

dPeripheral.1 = (Q2/V1 · Central) – (Q2/V2 · Peripheral.1)

dPeripheral.2 = (Q3/V1 · Central) – (Q3/V3 · Peripheral.2)

dPeripheral.3 = (Q4/V1 · Central) – (Q4/V4 · Peripheral.3)

dAbs.med = - (Kmed · Abs.med)

dElim = (CL/V1 · Central)

Model_NaL (inhaled insulin, Sakagami et al. (4))

dCentral = -(CL/V1 · Central) + (Kmed · Abs.med) - (Q2/V1 · Central) + (Q2/V2 · Peripheral.1) - (Q3/V1 · Central) + (Q3/V3 · Peripheral.2) - (Q4/V1 · Central) + (Q4/V4 · Peripheral.3)

dPeripheral.1 = (Q2/V1 · Central) – (Q2/V2 · Peripheral.1)

dPeripheral.2 = (Q3/V1 · Central) – (Q3/V3 · Peripheral.2)

dPeripheral.3 = (Q4/V1 · Central) – (Q4/V4 · Peripheral.3)

dAbs.med = - (Kmed · Abs.med) - (Knal · Abs.med)

dElim = (CL/V1 · Central) + (Knal · Abs.med)

Model_Transit (PF-00610355, Diderichsen et al. (5))

dCentral = -(CL/V1 · Central) + (Kmed · Abs.med) - (Q2/V1 · Central) + (Q2/V2 · Peripheral.1) - (Q3/V1 · Central) + (Q3/V3 · Peripheral.2) – (Q4/V1 · Central) + (Q4/V4 · Peripheral.3)

dPeripheral.1 = (Q2/V1 · Central) – (Q2/V2 · Peripheral.1)

dPeripheral.2 = (Q3/V1 · Central) – (Q3/V3 · Peripheral.2)

dPeripheral.3 = (Q4/V1 · Central) – (Q4/V4 · Peripheral.3)

dAbs.fast = - (Ktransit · Abs.fast)

dAbs.med = (Ktransit · Abs.fast) – (Kmed · Abs.med)

dElim = (CL/V1 · Central)

For ‘Model Transit’, dosing was performed into the ‘Abs.fast’ compartment, with transition from ‘Abs.fast’ to ‘Abs.med’ representing the transit process rather than parallel absorption.

S2.2. Parameter values used for simulation

Table S2. Parameter values used for data simulation. For abbreviations, see Table S1.

Parameter	Unit	I	Transit	NaL	II	IIIa
CL	[L/h]	46.0	1.40	24.6	44.7	74.2*
V1	[L]	15.0	17.8	4.40	11.8	23.5
Q2	[L/h]	17.4	16.0	81.8	9.97	31.7
V2	[L]	245	221	11.4	707	2590
Q3	[L/h]	0	0	0	55.0	65.7
V3	[L]	1#	1#	1#	40.4	473
Q4	[L/h]	0	0	0	12.5	22.5
V4	[L]	1#	1#	1#	103	16.1
F_{pul}	%	10.0%	77.2%	10.0%	49.0%	49.5%
PF1		-	-	-	0.383	0.701
PF2		-	-	-	-	0.889
K_{slow}	[h^{-1}]	-	-	-	1.18	0.0318
K_{med}	[h^{-1}]	0.180	0.852	0.0230	-	0.347
K_{fast}	[h^{-1}]	-	-	-	49.6	2.59
K_{NaL}	[h^{-1}]	-	-	1.09	-	-
$K_{Transit}$	[h^{-1}]	-	1.08	-	-	-
**ADDITIONAL PARAMETERS FOR EVALUATION OF PERFORMANCE ON CLINICAL DATASETS**						
$Prop_{iv,plasma}$	%CV	-	-	-	15.1	15.8
$Prop_{iv,urine}$	%CV	-	-	-	-	37.7
$Prop_{inh,plasma}$	%CV	-	-	-	15.5	15.8
$Prop_{inh,urine}$	%CV	-	-	-	-	37.7
IIV F_{Pul}	%CV	-	-	-	44.1	32.2 (IOV)
IIV CL	%CV	-	-	-	15.1	26.8 (CL_{NR})*
IIV V1	%CV	-	-	-	53.3	26.2
IIV Q2	%CV	-	-	-	18.9	25.7

* The original Model IIIa included two separate clearance values (renal and non-renal clearance, CL_R and CL_{NR}). The naïve-pooled analysis did not distinguish between these clearances; however, as interindividual variability (IIV) was put on the non-renal part, both clearances were implemented as separate processes in the population PK analysis. The respective values for CL_R and CL_{NR} were 10.5 and 63.7 L/h, respectively.

# For models with less than four systemic PK compartments originally (Models I, NaL, and Transit), Q values for the missing compartments (3 and 4) were set to 0 to remove drug transfer to these compartments while still allowing for automation of the simulation/re-estimation process. The corresponding Volumes of distribution were set to 1 to avoid division by 0.

Table S3. Initial base parameters used for estimation in the naïve-pooled analysis.

Parameter	Unit	I	Transit	NaL	II	IIIa
F_{pul}	%	50.0	50.0	50.0	50.0	50.0
PF1		-	-	-	0.500	0.500
PF2		-	-	-	-	0.500
K_{slow}	[h^{-1}]	-	-	-	0.100	0.0100
K_{med}	[h^{-1}]	0.100	0.100	0.100	-	0.100
K_{fast}	[h^{-1}]	-	-	-	1.00	1.00
K_{NaL}	[h^{-1}]	-	-	0.100	-	-
$K_{Transit}$	[h^{-1}]	-	0.100	-	-	-

Initial parameters for parallel retries were varied randomly using the *rnorm* function in R (random sampling from a specified normal distribution) as follows, resulting in a lognormal distribution of parameters:

$$Initial\ parameter = Initial\ base\ parameter \cdot e^{rnorm(n=1,\ mean=0,\ sd=1)} \tag{S4}$$

F_{Pul}, PF1 and PF2 were logit transformed beforehand to constrain the values between 0 and 1.

S2.3. Semi-mechanistic model

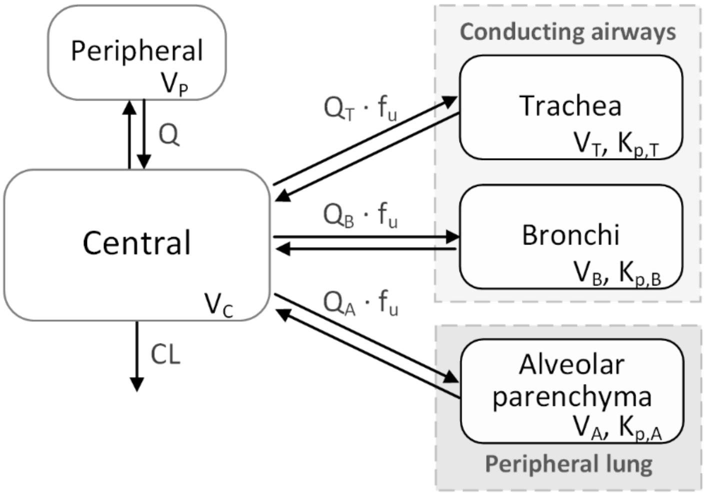

Figure S1. Structure of the semi-mechanistic model. CL: systemic clearance, V_C: central volume of distribution (Vd), Q: intercompartmental clearance, V_P: peripheral Vd; Q_T, Q_B, and Q_A represent the blood flow to the trachea, bronchi and alveolar parenchyma, respectively. f_u: fraction unbound in plasma, V_T: weight of the trachea, V_B: weight of the bronchi, V_A: weight of the alveolar parenchyma. $K_{p,T}$, $K_{p,B}$, and $K_{p,A}$ denote the tissue-to-plasma partition coefficients for the respective tissues. Adapted from Himstedt et al. (6).

The semi-mechanistic model was adapted to oral inhalation. The assumed pulmonary availability was 50%, with 80% of the lung dose depositing in the peripheral lung (alveolar parenchyma). The remaining 20% were equally distributed between the trachea and bronchi.

Table S4. Parameters used for simulation with the semi-mechanistic model. Abbreviations: see the caption of Figure S1.

Parameter	Unit	Value
CL	L/h/kg	0.971
V_C	L/kg	0.123
Q	L/h/kg	0.815
V_P	L/kg	3.77
f_u		0.014
K_T		6.52
K_B		18.6
K_A		39.3
Q_T	L/h/kg	0.0227
V_T	L/kg	0.0002
Q_B	L/h/kg	0.326
V_B	L/kg	0.000800
Q_A	L/h/kg	4.45
V_A	L/kg	0.00400

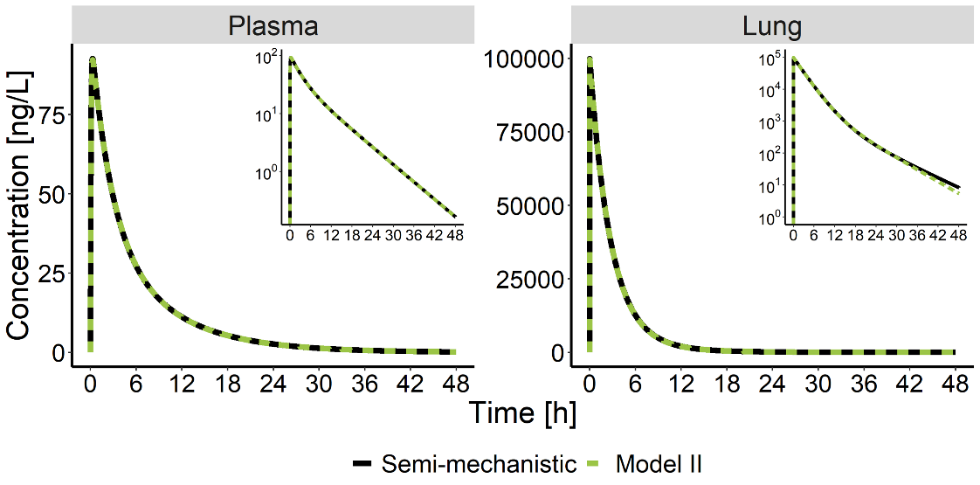

Figure S2. Plasma and lung concentration-time profiles. Simulation model: Semi-mechanistic lung distribution model (solid line). Estimation model: Model II (dashed line).

S3. Exemplary Figures: Scenarios 1 and 3

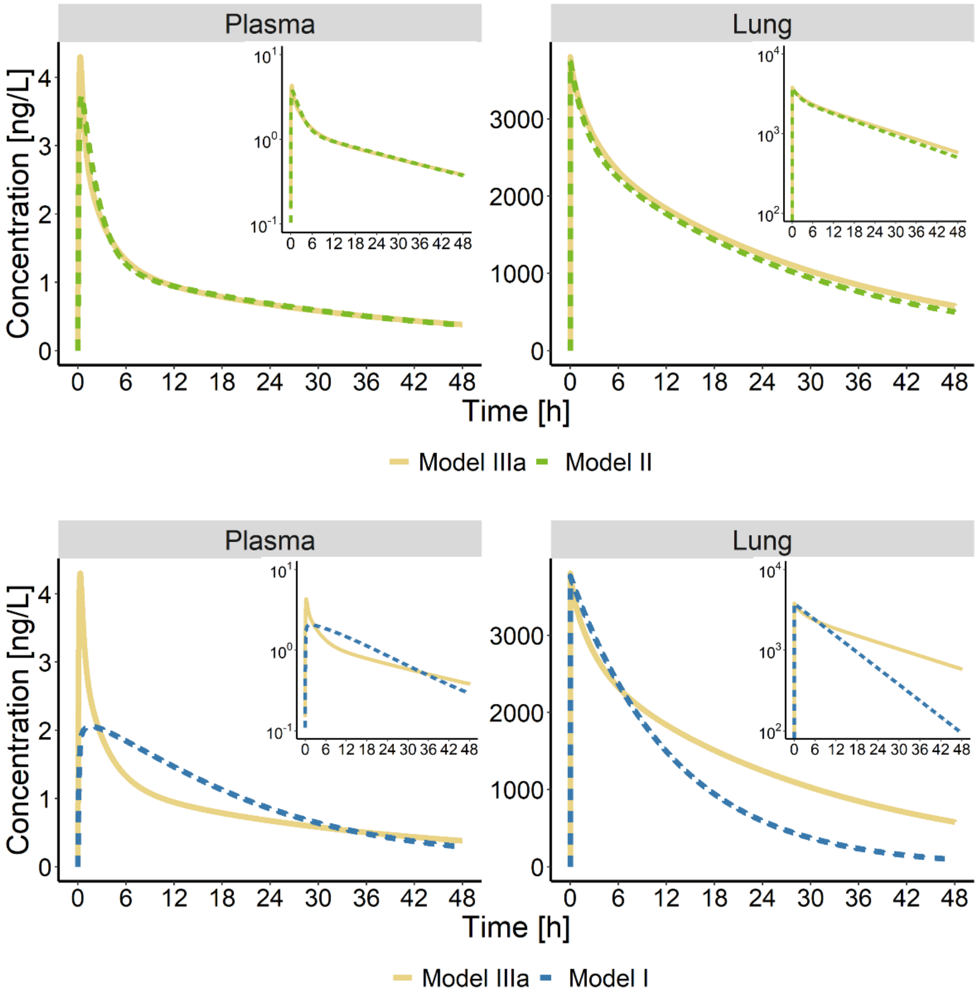

Figure S3. Exemplary plasma (left) and lung (right) concentration-time profiles for scenarios 1 (top) and 3 (bottom). Solid lines: Model used for simulation. Dashed lines: Predictions based on estimated model parameters and the respective model used for re-estimation.

S4. Non-compartmental analysis

The $AUC_{0\text{-last}}$ in plasma, as well as the area under the first moment curve ($AUMC_{0\text{-last}}$), were calculated via the log-linear trapezoidal method after both intravenous administration and oral inhalation. Extrapolation of the AUC to infinity was performed by addition of the last observed concentration divided by the terminal slope of log-transformed concentration data (C_{last}/λ_z). λ_z was determined by linear regression over the last three observations. The $AUMC_{0\text{-last}}$ was extrapolated to infinity by addition of the term $((C_{last} \cdot t_{last})/\lambda_z + C_{last}/\lambda_z^2)$, t_{last} denoting the time of the last observed concentration. Pulmonary bioavailability (F_{Pul}) was calculated as shown in Eq. S5:

$$F_{Pul} = \frac{AUC_{inhaled}}{AUC_{i.v.}} \cdot \frac{Dose_{i.v.}}{Dose_{inhaled}} \qquad \text{(S5)}$$

Inferring on pulmonary AUC ($AUC_{0\text{-inf,Lung}}$) was performed as follows: The $AUC_{0\text{-inf,plasma}}$ and $AUMC_{0\text{-inf,plasma}}$ were used to determine the mean residence time (MRT) for both administration routes:

$$MRT_{i.v.} = \frac{AUMC_{i.v.}}{AUC_{i.v.}} - \frac{T_{inf}}{2} \qquad \text{(S6)}$$

$$MRT_{inhaled} = \frac{AUMC_{inhaled}}{AUC_{inhaled}} \qquad \text{(S7)}$$

T_{inf} denotes the duration of the intravenous infusion.

The mean absorption time (MAT) was calculated by subtracting the mean residence time (MRT) after inhalation from the MRT after intravenous administration:

$$MAT = MRT_{inhaled} - MRT_{i.v.} \qquad \text{(S8)}$$

The MAT was then used to infer on the pulmonary absorption rate constant k_a:

$$k_a = \frac{1}{MAT} \qquad \text{(S9)}$$

To infer on the pulmonary $AUC_{0\text{-inf}}$, the equation for AUC calculation in plasma (Eq. S11) was adjusted to the lung, inserting F_{Pul} as the bioavailability (F) and the pulmonary absorption rate k_a as the elimination rate from the lung:

$$AUC_{0-Inf} = \frac{Dose \cdot F}{CL} \qquad \text{(S10)}$$

$$AUC_{0-inf,Lung} = \frac{Dose_{inhaled} \cdot F_{Pul}}{k_a \cdot V_{Lung}} \qquad \text{(S11)}$$

V_{Lung} was set to 0.840 L based on literature values for lung weight (7).

These analyses, performed in parallel to the population PK analyses, yielded ambivalent results for both scenarios. While the NCA performed on the dataset simulated with 'Model IIIa' resulted in plausible, yet biased values, the simulation with 'Model II' could not be analyzed with an NCA, as for some individuals the MRT after inhalation was shorter than after i.v. administration. In depth evaluation of the data indicated that this was due to biased $AUMC_{tz\text{-Inf}}$ values, i.e., the extrapolated area of the AUMC was underestimated compared to the true area. In agreement, analysis of inhalation PK data from individuals with negative MAT values showed that the terminal slope λ_z was overestimated (i.e. a steeper terminal profile was assumed, Figure S4) compared to the true value. For

this reason, and as this specific terminal part of the AUMC often constitutes a substantial part of the $AUMC_{0\text{-}Inf}$ (33), these individuals were characterized by an underestimated $AUMC_{inhaled}$. Combined with sometimes overestimated $AUMC_{0\text{-}inf,i.v.}$ values, this can explain the finding of negative MAT values. Thus, NCA for drugs with long terminal half-lives may necessitate even longer observation times or more accurate bioanalysis to adequately capture the terminal phase of the concentration-time profiles. This however might not always be feasible. Even for individuals with a positive MAT, the mean predicted $AUC_{0\text{-}inf,lung}$ was over tenfold higher than the true value. Furthermore, an NCA is only applicable if the same assumptions hold true as for the parallel absorption models, i.e. MCC and pulmonary metabolism being negligible (26). This leads to the conclusion that, the PK modeling approaches are more robust towards non-optimally designed sampling schemes, as well as providing more reliable estimates for the duration of pulmonary retention.

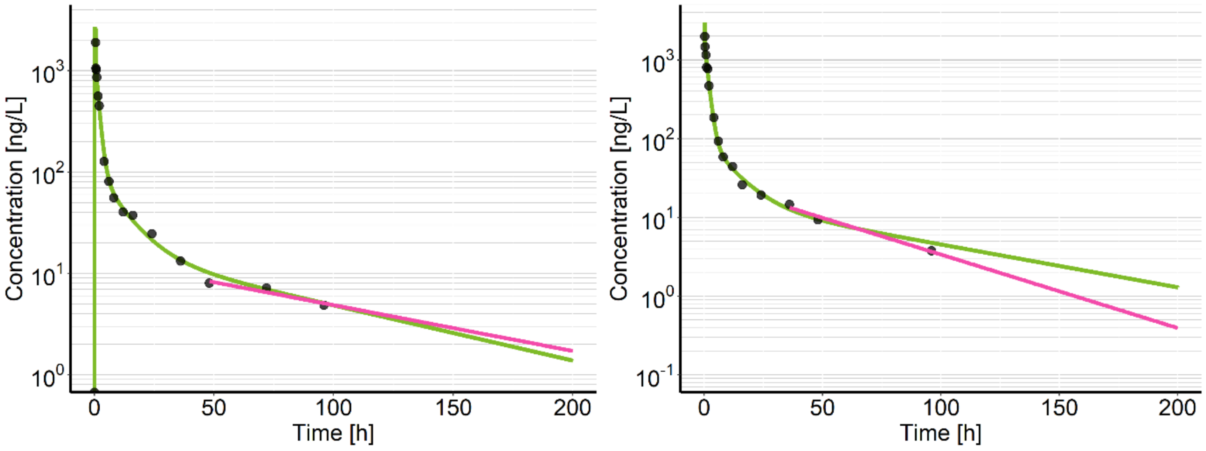

Figure S4. Deviation of true and calculated terminal slopes for an example individual after intravenous administration (left panel), and oral inhalation (right panel). Green line: true plasma concentration-time profile simulated with 'Model II'; Black dots: simulated "observed" data; Pink line: Extrapolation from the last three data points by linear regression. The combination of calculated slopes (too flat after intravenous dosing and too steep after inhalation) leads to negative MAT values for this individual, when calculated as described above.

S6. Comparison of parameter estimates between PPP, IPP, and ALL

Table S5. Median and 2.5th and 97.5th percentiles of PK parameters estimated the true model using three methods (PPP, IPP, and ALL). Simulation model: Model II/4CMT. For abbreviations, see Table S1

			Estimation method		
**Parameter**	**Unit**	**Simulation**	**PPP**	**IPP**	**ALL**
CL	[L/h]	44.7	44.7 [41.0, 48.7]	44.7 [41.1, 48.4]	44.8 [41.2, 48.7]
V1	[L]	11.8	11.7 [8.58, 16]	11.7 [8.74, 15.8]	11.6 [8.65, 15.8]
Q2	[L/h]	9.97	10.1 [8.77, 11.4]	10 [8.78, 11.4]	9.99 [8.9, 11.4]
V2	[L]	707	710 [575, 990]	706 [568, 999]	709 [614, 877]
Q3	[L/h]	55	55.4 [50.9, 61.1]	55.5 [50.9, 61.3]	55.3 [51.4, 60.1]
V3	[L]	40.4	40.5 [36.7, 44.3]	40.6 [36.7, 44.7]	40.4 [37.2, 44]
Q4	[L/h]	12.5	12.7 [11.4, 14.2]	12.7 [11.3, 14.1]	12.7 [11.8, 13.7]
V4	[L]	103	104 [85.8, 127]	104 [85.5, 126]	105 [90.8, 119]
$Prop_{iv}$	%CV	15.1	15.3 [14, 16.8]	15.3 [14, 16.7]	-
F_{pul}	%	49.0%	48.1% [41.5%, 56.3%]	48.9% [42.2%, 56.2%]	48.6% [42.3%, 55.9%]
PF1		0.383	0.399 [0.269, 0.536]	0.397 [0.301, 0.473]	0.405 [0.319, 0.484]
K_{slow}	[h^{-1}]	1.18	1.18 [0.964, 1.47]	1.19 [0.975, 1.49]	1.19 [0.985, 1.47]
K_{fast}	[h^{-1}]	49.6	63.3 [17.1, 98600]	45.4 [26.1, 207]	43.6 [24.6, 163]
$Prop_{inh}$	%CV	15.5	15.5 [13.8, 17.5]	15.5 [13.9, 17.1]	15.2 [13.9, 16.7]
IIV F_{Pul}	%CV	44.1	40.6 [18.8, 63.2]	40.9 [24.9, 57.7]	40.5 [25, 57.6]
IIV CL	%CV	15.1	13.9 [8.5, 19.6]	14.0 [8.7, 20.3]	13.9 [8.2, 19.6]
IIV V1	%CV	53.3	50.8 [29.3, 75.8]	50.6 [29.3, 74.1]	50.1 [29.6, 76.6]
IIV Q2	%CV	18.9	17.2 [7, 26.5]	17.7 [7.4, 26.8]	17.9 [9.2, 26.6]

1. Borghardt JM, Weber B, Staab A, Kunz C, Formella S, Kloft C. Investigating pulmonary and systemic pharmacokinetics of inhaled olodaterol in healthy volunteers using a population pharmacokinetic approach. British Journal of Clinical Pharmacology. 2016;81(3):538-52.
2. Melin J, Prothon S, Kloft C, Cleton A, Amilon C, Jorup C, et al. Pharmacokinetics of the Inhaled Selective Glucocorticoid Receptor Modulator AZD5423 Following Inhalation Using Different Devices. AAPS Journal. 2017;19(3):865-74.
3. Krishnaswami S, Hochhaus G, Möllmann H, Barth J, Derendorf H. Interpretation of absorption rate data for inhaled fluticasone propionate obtained in compartmental pharmacokinetic modeling. International Journal of Clinical Pharmacology and Therapeutics. 2005;43(3):117-22.
4. Sakagami M. Insulin disposition in the lung following oral inhalation in humans: A meta-analysis of its pharmacokinetics. Clinical Pharmacokinetics. 2004;43(8):539-52.
5. Diderichsen PM, Cox E, Martin SW, Cleton A, Ribbing J. Characterizing systemic exposure of inhaled drugs: application to the long-acting beta2-agonist PF-00610355. Clin Pharmacokinet. 2013;52(6):443-52.
6. Himstedt A, Braun C, Wicha SG, Borghardt JM. Towards a Quantitative Mechanistic Understanding of Localized Pulmonary Tissue Retention—A Combined In Vivo/In Silico Approach Based on Four Model Drugs. Pharmaceutics. 2020;12(5):408.
7. Molina DK, DiMaio VJM. Normal Organ Weights in Men: Part II—The Brain, Lungs, Liver, Spleen, and Kidneys. The American Journal of Forensic Medicine and Pathology. 2012;33(4).
